# Changes in Nutritional Metabolites of Young Ginger (*Zingiber officinale* Roscoe) in Response to Elevated Carbon Dioxide

**DOI:** 10.3390/molecules191016693

**Published:** 2014-10-16

**Authors:** Ali Ghasemzadeh, Hawa Z. E. Jaafar, Ehsan Karimi, Sadegh Ashkani

**Affiliations:** 1 Department of Crop Science, Faculty of Agriculture, University Putra Malaysia, 43400 Serdang, Selangor, Malaysia; E-Mails: ehsan_b_karimi@yahoo.com; 2 Institute of Tropical Agriculture, University Putra Malaysia, 43400 Serdang, Selangor, Malaysia; 3 Department of Agronomy and Plant Breeding, Shahr-e-Rey Branch, Islamic Azad University, Tehran, Iran; E-Mail: ashkani.sadegh@upm.edu.my

**Keywords:** sugars, CO_2_ enrichment, protein, amino acids, antinutrient, *Zingiber officinale*

## Abstract

The increase of atmospheric CO_2_ due to global climate change or horticultural practices has direct and indirect effects on food crop quality. One question that needs to be asked, is whether CO_2_ enrichment affects the nutritional quality of Malaysian young ginger plants. Responses of total carbohydrate, fructose, glucose, sucrose, protein, soluble amino acids and antinutrients to either ambient (400 μmol/mol) and elevated (800 μmol/mol) CO_2_ treatments were determined in the leaf and rhizome of two ginger varieties namely Halia Bentong and Halia Bara. Increasing of CO_2_ level from ambient to elevated resulted in increased content of total carbohydrate, sucrose, glucose, and fructose in the leaf and rhizome of ginger varieties. Sucrose was the major sugar followed by glucose and fructose in the leaf and rhizome extract of both varieties. Elevated CO_2_ resulted in a reduction of total protein content in the leaf (H. Bentong: 38.0%; H. Bara: 35.4%) and rhizome (H. Bentong: 29.0%; H. Bara: 46.2%). In addition, under CO_2_ enrichment, the concentration of amino acids increased by approximately 14.5% and 98.9% in H. Bentong and 12.0% and 110.3% in H. Bara leaf and rhizome, respectively. The antinutrient contents (cyanide and tannin) except phytic acid were influenced significantly (*P* ≤ 0.05) by CO_2_ concentration. Leaf extract of H. Bara exposed to elevated CO_2_ exhibited highest content of cyanide (336.1 mg HCN/kg DW), while, highest content of tannin (27.5 g/kg DW) and phytic acid (54.1 g/kg DW) were recorded from H.Bara rhizome grown under elevated CO_2_. These results demonstrate that the CO_2_ enrichment technique could improve content of some amino acids and antinutrients of ginger as a food crop by enhancing its nutritional and health-promoting properties.

## 1. Introduction

Environmental conditions, cultivation practices, and management approaches can impact the quality of foods and their abilities to promote good health and well-being. Indeed, new management strategies using ecophysiological conditions to promote phytochemical content in food crops are starting to be implemented. A number of ecophysiological conditions, including environmental, cultivation conditions and management practices such as daily temperature, daily irradiation, fertilizer supply, irrigation, and production time are considered to significantly enhance the levels of health-promoting compounds in vegetable crops. Accordingly, there is rising interest in improving strategies and management practices in order to enhance the nutrition and health-promoting properties of food crops.

CO_2_ enrichment is one of the techniques that has recently been used around the world to enhance the qualitative and quantitative yield of plants [1,2]. The mechanisms by which plants adapt to changeable environmental conditions are a most important scientific challenge. The CO_2_ concentration in the atmosphere is steadily increasing, which affects plant morphogenesis, physiology and photosynthesis. There are currently some general models [3,4,5] to predict plant adaptation to a varied environment, because the response to rising of CO_2_ concentration is dependent on plant development stage and species and can be modified by a number of factors, including water availability, light and nutrient [6]. Exposure of plants to elevated CO_2_ conditions in general enhances photosynthesis rates due to the induced Rubisco enzyme activity [7] and following that, production of primary and secondary metabolites were enhanced [8]. Ibrahim and Jaafar [9] reported that the highest net photosynthesis was obtained in *Labisia pumila* exposed to elevated CO_2_ (1200 μmol/mol) conditions. There is accumulating evidence to suggest that many crops show increasing photosynthetic rates, growth rates, and primary and secondary metabolite productions in response to increased carbon dioxide content [10,11,12,13]. The results of recent studies by the current authors showed that the anticancer and antioxidant activity of ginger was enhanced in response to elevated CO_2_ [14].

Carbohydrates (soluble and insoluble) are the most important components in plants and many food crops and contribute to their nutritive value [15]. Carbohydrates may be chemically bound to other molecules or physically associated or present as isolated molecules [2,8]. A recent study has revealed that during growth under elevated CO_2_ levels, the leaf starch content increased on average by 160% and soluble carbohydrate content by 52% [7]. However, these rapid changes are having a serious effect on plant growth and development. This concept has recently been challenged by recent studies demonstrating that enhancement of carbohydrates in the leaf resulted in inhibition of photosynthesis rate due to a reduction of Rubisco enzyme activity, which is responsible for carbon dioxide fixation [16,17]. CO_2_ enrichment generates increased sucrose content in the leaf and intense hydrolytic decomposition into fructose and glucose through acid invertase [8,18,19,20].

At present, in Malaysia ginger varieties (H. Bentong and H. Bara) are mainly cultivated along the steep sloped lands of Bentong village in Pahang and Tambunan province. The manipulation of cultivation techniques for good yield and pharmacological quality of young ginger is still not fully established and need to be considered critically, especially in terms of enhancing its nutritional quality in terms of protein, free amino acids, carbohydrate and antinutrient content. So far, however, there has been little discussion of the role of carbon dioxide in the production and synthesis of free amino acids in Malaysian young ginger varieties. Furthermore, information on the effects of elevated carbon dioxide on the antinutrient and amino acids content of Halia Bentong and Halia Bara is not available. The aim of this study was therefore to determine changes in the synthesis of carbohydrates, amino acids and antinutrients in the leaf and rhizome of H. Bentong and H. Bara as influenced by CO_2_ levels.

## 2. Results and Discussion

### 2.1. Effect of Ambient and Elevated CO_2_ on Total Carbohydrate and Protein Content

Total carbohydrate levels were influenced by CO_2_ level (Table 1; *P* ≤ 0.01). In general, the highest value of total carbohydrates was observed in the rhizome, followed by leaf. In the plants exposed to elevated CO_2_ levels (800 μmol/mol), the production of total carbohydrate was enhanced. High content of total carbohydrate was observed in H. Bentong rhizome, with a value of 288.4 mg/g DW under elevated CO_2_ conditions. The results of previous studies have revealed that elevated CO_2_ conditions enhanced soluble sugar content of *Labisia pumila* Blume [9], *Urtica diocia* and *Plantago major* [21], *Poa alpinia* [22] and beech leaf by about 52% [23]. CO_2_ enrichment often enhances the concentration of total carbohydrates and possibly stimulates the metabolism of secondary metabolites in plants [8]. With an increase in CO_2_ concentration from ambient to elevated concentration, the total carbohydrate content was enhanced by 41.10% and 63.39% in the leaf and rhizome of H. Bentong, respectively, and by 34.94% and 45.48% in the leaf and rhizome of H. Bara, respectively. Increasing the CO_2_ concentration from 400 to 800 μmol/mol resulted in a reduction in total protein content in the rhizome and leaf. As can be seen from the Table 1, the content of total protein in the ginger varieties was higher in the rhizome than in the leaf. A high content of total protein was recorded in the rhizome extract of H. Bara (27.5 mg/g DW) when grown under ambient CO_2_ conditions. Furthermore, the total protein content decreased by 38.0% and 29.9% in the leaf and rhizome of H. Bentong, respectively, and by 35.4% and 46.2% in the leaf and rhizome of H. Bara, respectively. Taub *et al.* [24] showed that by increasing the CO_2_ level from ambient to elevated the protein content of barley, wheat, and rice grains decreased by 10% to 15%. In addition, the high CO_2_ concentration enhanced the reduction in tuber protein concentration in potato by about 14%. Högy and Fangmeier [25] reported protein content of potato decreased when exposed to elevated CO_2_. More importantly, content and type of protein of plant could be altered due to the CO_2_ enrichment technique, as shown for wheat [25] and rice [26]. Two phytocentric conceptual models have been advanced to explain the effects of elevated CO_2_, on carbon partitioning in plant parts to secondary metabolites: the growth/differentiation balance hypothesis (GDBe) [27], and the protein competition model (PCM), [28]. Also there is a developmental systems model, but strictly focused around a particular mechanism, and accordingly it makes more exact predictions about how elevated CO_2_ effects on source/sink interactions impact partitioning to proteins and amino acids. The reduction in protein concentration of ginger is partially could be due to enhanced carbon assimilation and constrained N uptake under elevated CO_2_. Overall, these changes (reduction of proteins) result in adjustments in the C/N ratios of leaves [29].

**Table 1:** Total carbohydrates and protein content in two variety of ginger grown under different CO_2_ concentration (400 and 800 μmol/mol).

Variety	Part	Total Carbohydrate		Total Protein	
		400	800	400	800
H. Bentong	Leaf	142.80 ± 12.44 ^e^	201.50 ± 11.62 ^c^	16.30 ± 1.51 ^d^	11.80 ± 1.46 ^e^
	Rhizome	176.50 ± 11.85 ^d^	288.40 ± 13.13 ^a^	21.70 ± 1.56 ^b^	16.70 ± 1.75 ^d^
H. Bara	Leaf	140.50 ± 11.63 ^e^	189.60 ± 11.92 ^c^	16.80 ± 1.33 ^d^	12.40 ± 1.92 ^e^
	Rhizome	168.20 ± 11.41 ^d^	244.70 ± 11.81 ^b^	27.50 ± 1.87 ^a^	18.80 ± 1.38 ^c^

Notes: Data are means of triplicate measurements ± standard deviation. Means not sharing a common single letter for each measurement were significantly different at *P* ≤ 0.05. Unit of total carbohydrate and protein: mg/g DW.

### 2.2. Effect of Ambient and Elevated CO_2_ on Sugars (Sucrose, Glucose and Fructose) Content

The concentration of sucrose, glucose, and fructose were influenced significantly (*P* ≤ 0.05) by CO_2_ concentration. Ginger varieties grown under 400 μmol/mol CO_2_, showed the highest value of these sugars in the rhizome compared to the leaf. Under ambient conditions, the concentration of sucrose in the leaf and rhizome of the two varieties was highest, followed by glucose and fructose (Table 2). When comparing the leaf extract of H. Bentong and H. Bara grown under the 400 μmol/mol CO_2_, it was found that H. Bentong had higher sucrose, glucose and fructose contents than H. Bara. Under ambient CO_2_ condition, rhizome extract of H. Bentong represent higher content of sucrose and lower content of glucose and fructose compared to H. Bara. Behboudian and Tod [30] reported that elevated CO_2_ enhanced the quality of tomato by increasing their soluble carbohydrate (sucrose, glucose and fructose) concentrations. The leaves of both varieties present higher increases in all soluble sugar contents at 800 μmol/mol CO_2_ compared to 400 μmol/mol CO_2_ (Table 2). Among the studied sugars, percentage enhancement of fructose was higher compared to sucrose and glucose. As shown in Table 2, the increase in carbohydrate content was greater in rhizome than in leaves under elevated CO_2_ conditions. Increases in sucrose and glucose under enrichment conditions have also been reported in other plants such as orchids, sugarcane, tomatoes and potatoes [25,31,32]. Increases in sucrose and glucose could be due to an increase in hexose phosphate synthesis under high CO_2_ concentrations. Hexose phosphate is a progenitor for sucrose synthesis, therefore, as hexose phosphate concentration increases, the synthesis of glucose and sucrose is concomitantly enhanced [33]. Contrary to our result, Demmers-Derks *et al.* [34] reported that elevated CO_2_ did not change sucrose levels in *Beta vulgaris*. The results of current study consistent with De Souza *et al.* [32] who indicated that sucrose concentration increased about 29% in sugar cane grown under elevated CO_2_ conditions (740 ppm). Norby *et al.* [35] showed that elevated CO_2_ could enhance sucrose concentration in roots and simplify the mobilization of nitrogen and carbon compounds to new primordial roots.

**Table 2:** Sucrose, glucose and fructose content in two variety of ginger grown under different CO_2_ concentration (400 and 800 μmol/mol).

Variety	Part	Sucrose		Glucose		Fructose	
		400	800	400	800	400	800
H. Bentong	Leaf	25.00 ± 1.84 ^d^	38.20 ± 1.42 ^b^	10.60 ± 1.77 ^f^	27.20 ± 2.44 ^c^	7.40 ± 0.94 ^e^	15.60 ± 1.62 ^c^
	Rhizome	33.50 ± 1.19 ^c^	44.60 ± 2.31 ^a^	17.40 ± 1.38 ^e^	31.50 ± 1.82 ^b^	8.60 ± 1.33 ^d^	21.30 ± 2.71 ^a^
H. Bara	Leaf	20.60 ± 1.65 ^e^	37.50 ± 1.78 ^b^	6.30 ± 0.95 ^g^	22.60 ± 1.69 ^d^	5.90 ± 1.74 ^f^	19.40 ± 1.18 ^b^
	Rhizome	27.60 ± 1.22 ^d^	41.80 ± 1.49 ^a^	18.60 ± 1.29 ^e^	36.20 ± 1.28 ^a^	9.50 ± 2.52 ^d^	22.10 ± 2.66 ^a^

Notes: Data are means of triplicate measurements ± standard deviation; Means not sharing a common single letter for each measurement were significantly different at *P* ≤ 0.05; Unit of all measurement are mg/g DW.

### 2.3. Effect of Ambient and Elevated CO_2_ on Amino Acids Content in Ginger

The results indicated that, rising of CO_2_ concentration from ambient (400 μmol/mol) to elevated (800 μmol/mol) resulted in enhanced levels of most amino acids in the leaf and rhizome of ginger varieties. Amino acid content also influenced significantly (*P* ≤ 0.05) by ginger variety and plant part. Among the studied amino acids, histidine and lysine were not detected from H. Bentong and H. Bara rhizome grown under 400 μmol/mol CO_2_ (Table 3). The total soluble amino acids concentration in H. Bara grown under ambient CO_2_ treatment (leaf: 209.9 µmol/g DW; rhizome: 165 µmol/g DW) was higher than in H. Bentong (leaf: 185.2 µmol/g DW; rhizome: 123.6 µmol/g DW). Similarly, under elevated CO_2_ conditions the total essential amino acids of H. Bara (leaf: 235.2 µmol/g DW; rhizome: 347.1 µmol/g DW) was higher than in H. Bentong (leaf: 212.1 µmol/g DW; rhizome: 245.9 µmol/g DW). In addition, in response to elevated CO_2_ the concentration of amino acids increased by approximately 14.5% and 98.9% in H. Bentong leaf and rhizome, respectively. In H. Bara the concentration of soluble amino acids also increased by approximately 12.0% and 110.3% in the leaf and rhizome under 800 μmol/mol CO_2_, respectively. Under a CO_2_ concentration of 400 μmol/mol, the amount of glutamine, histidine, leucine, valine, and tyrosine in leaf extracts were higher than rhizome extracts. Furthermore, under ambient condition (400 μmol/mol) histidine was not detected from a rhizome extract of both varieties. Additionally, lysine was not detected from leaf extract of H. Bentong grown under 400 μmol/mol CO_2_. The most abundant soluble amino acids in H. Bentong were glutamine, and glutamic acid, while, in H. Bara were leucine. The present finding that increasing of soluble amino acids by CO_2_ enrichment in young ginger leaf is in agreement with previous reports in soybean [36], tobacco [37], barley [38] and cotton [39]. Alcohol-soluble fractions principally contain carbohydrates, organic acids and amino acids, respectively. It is widely agreed that plant growth in CO_2_ enriched atmospheres enhances the accumulation of both leaf starch and soluble carbohydrates [32,35]. Since the metabolism of carbohydrates is essential for the synthesis of amino acids, it is reasonable to assume that the effects of CO_2_ enrichment should be similar for these classes of compounds [19]. Ample carbon was available to support amino acid synthesis and the increase in soluble amino acids under CO_2_ enrichment [19]. In the current study, the relationship between protein and amino acids content is interesting because with decreasing of protein content the amount of free amino acids increased. Amino acids are the basic structures of proteins and each type of protein depends on the arrangement of the amino acids. Increasing amino acids content in the current study could be related to degradation of proteins under elevated CO_2_ conditions and hydrolysis to free amino acids [40]. In general, protein synthesis is balanced by an equal amount of protein degradation. Further experimental investigations are needed to estimate relationship between CO_2_ enrichment and free amino acids productions in crops.

### 2.4. Effect of Ambient and Elevated CO_2_ on Antinutrient Content

The results of antinutrient content in the leaf and rhizome of H. Bentong and H. Bara exposed to 400 and 800 μmol/mol CO_2_ are presented in Table 4. As shown in this table, antinutrient contents (cyanide and tannin) except phytic acid were influenced significantly (*P* ≤ 0.05) by CO_2_ concentration in both varieties. The cyanide content in the leaf and rhizome was enhanced when the plants were exposed to elevated CO_2_. The percentage increases in cyanide in ginger when exposed to elevated CO_2_ condition in the leaf and rhizome of H. Bentong were 89% and 17%, respectively, and 78% and 39.9%, respectively, in H. Bara. A high content of cyanide (336.1 mg HCN/kg DW) was recorded in H. Bara leaf grown under 800 μmol/mol CO_2_. Cyanide was the most concentrated antinutrient in the two ginger varieties. Accumulation and partitioning of cyanide in the leaf and rhizome of ginger followed the trend leaf > Rhizome. Cyanides are familiar to us as the taste of bitter almonds and is a toxin produced by some plants to dissuade potential consumers. A number of studies suggest that the toxicity of some crops will increase with rising CO_2_ levels because of enhancing of some bioactive compounds and antinutrients like cyanide [41]. Further research should be done to investigate the effect of CO_2_ enrichment on toxicity of H.Bentong and H.Bara. It was found that CO_2_ enrichment had a significant effect on the tannin content of both varieties. The maximum tannin content (27.5 g/kg DW) was observed in extract of H. Bara rhizome grown under 800 μmol/mol CO_2_ (Table 4). What is interesting in this study is that tannin was not found in ginger grown under 400 μmol/mol CO_2_. Rising of CO_2_ level from ambient to elevated resulted in enhancement of tannin in the leaf and rhizome. Our result is consistent with Mattson *et al.* [5] who reported that under elevated CO_2_ tannins content enhanced by approximately 8%, 15% and 37% in root, stem and leaf of birch seedlings, respectively. Oksanen *et al.* [42] and Lindroth *et al.* [43] have also found that CO_2_ enrichment enhanced tannin and starch content in the paper birches. No significant difference was observed between ambient and elevated CO_2_ for phytic acid content in those varieties. A high value of phytic acid (54.1 g/kg DW) was recorded in the rhizome of H. Bara rhizome grown under elevated CO_2_ concentration. Phytic acid content decreased in the leaf extract of those varieties with increasing of CO_2_ concentration, while, in the rhizome extract increased. Phytic acid may be considered a phytonutrient, providing an antioxidant effect, and is the principal storage forms of phosphorus in plants [44]. Information on the CO_2_ enrichment effects on the production of phytic acid on ginger varieties is not available, making the information of the current study useful for future studies.

**Table 3:** Amino acids content in two variety of ginger grown under different CO_2_ concentration (400 and 800 μmol/mol).

	H. Bentong				H. Bara			
	Leaf		Rhizome		Leaf		Rhizome	
	400	800	400	800	400	800	400	800
Glutamine	41.30 ± 1.17 ^a^	36.10 ± 2.14 ^ab^	20.10 ± 1.53 ^c^	37.00 ± 1.27 ^ab^	22.40 ± 2.18 ^c^	33.20 ± 1.18 ^b^	14.80 ± 0.88 ^d^	31.70 ± 2.06 ^b^
Histidine	20.10 ± 1.44 ^b^	27.50 ± 1.48 ^a^	ND	12.50 ± 1.14 ^d^	12.50 ± 1.38 ^d^	15.30 ± 1.22 ^c^	ND	6.30 ± 0.36 ^e^
Glutamic acid	28.10 ± 1.73 ^d^	37.20 ± 1.73 ^c^	28.80 ± 1.03 ^d^	53.10 ± 2.16 ^a^	16.40 ± 0.86 ^f^	27.50 ± 1.32 ^d^	22.50 ± 1.16 ^e^	43.70 ± 1.44 ^b^
Threonine	16.40 ± 1.93 ^f^	18.20 ± 1.66 ^f^	22.40 ± 1.55 ^e^	43.50 ± 1.28 ^b^	21.80 ± 1.22 ^e^	28.40 ± 1.25 ^d^	34.20 ± 2.18 ^c^	64.80 ± 2.83 ^a^
Leucine	37.50 ± 2.3 ^d^	28.40 ± 1.15 ^e^	22.10 ± 1.83 ^f^	40.70 ± 1.66 ^c^	42.70 ± 1.72 ^c^	54.70 ± 2.17 ^b^	41.50 ± 2.16 ^c^	88.90 ± 3.29 ^a^
Lysine	ND	4.20 ± 0.87 ^d^	2.80 ± 0.38 ^d^	5.50 ± 0.79 ^d^	13.60 ± 0.69 ^c^	20.50 ± 1.18 ^b^	14.40 ± 1.06 ^c^	29.40 ± 2.04 ^a^
Valine	29.40 ± 1.29 ^c^	41.10 ± 2.31 ^b^	17.20 ± 1.55 ^d^	31.70 ± 1.27 ^c^	32.50 ± 1.62 ^c^	51.60 ± 2.77 ^a^	19.40 ± 1.16 ^d^	41.60 ± 1.26 ^b^
Tyrosine	12.40 ± 1.17 ^d^	19.40 ± 1.66 ^c^	10.20 ± 1.73 ^d^	21.90 ± 1.06 ^c^	18.30 ± 1.17 ^c^	33.70 ± 2.19 ^b^	18.20 ± 1.29 ^c^	40.80 ± 1.18 ^a^
Total	185.20 ± 1.88 ^d^	212.10 ± 3.52 ^c^	123.60 ± 3.52 ^e^	245.90 ± 1.44 ^b^	209.90 ± 2.88 ^c^	235.20 ± 3.40 ^b^	165.00 ± 2.77 ^d^	347.10 ± 3.28 ^a^

Notes: Data are means of triplicate measurements ± standard deviation; Means not sharing a common single letter for each measurement were significantly different at *P* ≤ 0.05; ND: not detected; Units are µmol/g DW.

**Table 4:** Antinutrient contents in the leaf and rhizome of two ginger varieties grown under different CO_2_ concentration (400 and 800 μmol/mol).

	H. Bentong				H. Bara			
	Leaf		Rhizome		Leaf		Rhizome	
	400	800	400	800	400	800	400	800
Cyanide	117.5 ± 2.6 ^e^	223.1 ± 4.7 ^b^	87.4 ± 1.4 ^f^	102.7 ± 1.5 ^e^	188.2 ± 3.9 ^c^	336.1 ± 2.7 ^a^	92.4 ± 3.7 ^f^	129.3 ± 2.4 ^d^
Tannin	ND	ND	ND	22.5 ± 2.2 ^b^	ND	19.2 ± 1.1 ^c^	ND	27.5 ± 1.9 ^a^
Phytic acid	21.4 ± 1.3 ^d^	17.1 ± 3.9 ^d^	44.2 ± 2.6 ^b^	46.8 ± 2.8 ^b^	36.2 ± 1.7 ^c^	33.4 ± 1.2 ^c^	52.9 ± 3.2 ^a^	54.1 ± 2.3 ^a^

Notes: Data are means of triplicate measurements ± standard deviation; Means not sharing a common single letter for each measurement were significantly different at *P* ≤ 0.05; Cyanide: mg HCN/kg DW; Tanin: g/kg DW; Phytic acid: g/kg DW; ND: not detected.

## 3. Experimental Section

### 3.1. Plant Materials

Rhizome of two young ginger varieties (H. Bentong and H. Bara) were germinated in small pots for 14 days and then transferred to polyethylene bags which is filled with a mixture of coco peat and burnt rice husk (1:1). Seedlings with 2–3 leaf were transferred to three CO_2_ growth chamber (Conviron EF7, Winnipeg, MB, Canada) with two different CO_2_ concentrations (400 and 800 µmol/mol). The split plot experiment was designed with CO_2_ as a main plot and ginger varieties as a sub plot with three replications.

### 3.2. Growth Chamber Microclimate

The concentration and flow of CO_2_ to the chamber was controlled and monitored with a PPM_3_ Controller (Custom Automated Products, Nevark, NJ, USA). In chamber environmental conditions like temperature, relative humidity and light intensity were controlled using data management system software (Dynamac Corp., Rockville, MD, USA). The plants were irrigated with a super drip irrigation system. Chicken dung (30 g) was applied into the each media at the beginning of the experiment. Stock solution of nutrient (N, P and K) recommended by Ravindran and Nirmal was dissolved in irrigation water with ratio 1:20 every week. After four month, plants were harvested and rhizome and leaf were separated, washed and freeze dried. All samples were kept at −80 °C for future analysis.

### 3.3. Total Carbohydrate Content

Freeze dried samples (0.1 g) were extracted with 25 mL of ethanol (80%) and stored. Homogenized solutions were centrifuged (5000 rpm) and 1 mL of supernatant was added to anthrone solution (10 mL, 0.15%) and heated. Samples were cooled at room temperature for 10 min and then absorption of the samples was recorded at 625 nm [45].

### 3.4. Total Protein Content

A 500 mg sample of freeze dried leaf and rhizome were collected from each variety and treatment and protein was extracted in 3 mL of Tris-HCl buffer (0.10 mM Tris-HCl and 0.15 M NaCl) (pH 7.6). Samples were then centrifuged twice at 10,000 rpm at 4 °C for 30 min to get a clear supernatant for hydrophilic protein content mesearment. The pellet was subsequently re-suspended with 3 mL SDS-Tris-HCl buffer (0.10 Mm Tris-HCl, 0.15 M NaCl and 2% SDS) (pH 7.6) and spun and mixed for 1 h. Extracted suspensions were then centrifuged twice at 10,000 rpm at 4 °C for 30 min and supernatant was collected for hydrophobic protein content measurement [46]. Samples diluted five times and 100 µL of extraction was mixed with 3 mL of Coomassie G-250 reagent (4.7% ethanol, 8.5% phosphoric acid and 0.01% Coomassie brilliant blue G). Absorbance of samples were measured at 595 nm after 5 and 30 min of reaction using a spectrophotometer (U-2001, Hitachi Instruments Inc., Tokyo, Japan). Bovine serum albumin was used as a standard. Total protein content was the sum of salt soluble protein content and SDS soluble protein content.

### 3.5. High Performance Liquid Chromatography (HPLC) of Sugars

#### 3.5.1. Extraction

Plant powder (50 mg) was added to extraction solvent mixture (chloroform/methanol/water). In order to make this mixture first, a methanol/water solution (10 mL, 1:1 v/v) was added to samples and after that chloroform (3 mL) was added. The solution was homogenized using a vortexer and then stored at 4 °C for 30 min. After that, tubes were centrifuged (5500  rpm, 4 °C) for 15 min and about 8  mL of supernatant (methanol/water) was transferred to a 15 mL Falcon tube and after evaporation under vacuum, extracts were kept at −20 °C for carbohydrate analysis [47].

#### 3.5.2. HPLC Method

An Agilent HPLC 1200 Series, Refractive Index Detector (RID) with a ZORBAX NH_2_ Column, 5 μm, 250 × 4.6 mm, was used to identify and determine the soluble sugars in ginger. The sample extracts were filtered through a filter (0.45 μm, nylon type) and 10 μL of solution taken for injection with flow rate of 1.8 mL/min. Acetonitrile and water (75:25, v/v) were used as mobile phase. The standards of fructose, glucose, and sucrose were used in the concentration range of 1% to 5% (w/v). A calibration curve was obtained for each sugar. Glucose, sucrose and fructose standards were dissolved in water/acetonitrile (50:50, v/v) with different concentratione: 0.25, 0.5, 1, 2 and 4 mg/mL [48]. The linear regression equation were calculated with *Y* = *aX* ± *b*, where *X* was concentration of flavonoid and *Y* was the peak area of flavonoids obtained from HPLC. The linear regression equation for glucose, sucrose and fructose were: *Y* = 18842*X* + 162.7, 20837*X* − 17933.6 and *Y* = 21559*X* − 215.9 respectively.

### 3.6. Ultra High Performance Liquid Chromatography Analysis of Amino Acids

#### 3.6.1. Extraction

Plant material (0.1 g) was mixed with ethanol (5 mL, 70%) and sonicated for 30 min. Samples were centrifuged at 1500 rpm for 5 min and the supernatant decanted. The residue was extracted twice with 5 mL of the extraction solvent and decanted extracts was combined. The final volume was adjusted to 15.0 mL. An aliquot (2.0 mL) of the combined extracts was placed in a glass vial and heated at 60 °C and the solvent removed by evaporation under a flow of nitrogen. In order to evaporate any water in the samples about 1 mL absolute ethanol was added to each vial and the samples were then evaporated to dryness. Water, methanol, triethylamine and phenyl isothiocyanate were mixed with ratio of 80:10:5:5 and 0.25 mL of this solution was mixed with samples by mechanical rotation for 15 min. The samples were dried under nitrogen flow on the heat block (60 °C) and the dry residue was reconstituted in 1.0 mL of 50% acetonitrile/water and transferred to autosampler vials for analysis.

#### 3.6.2. UHPLC Method

A UHPLC instrument (Agilent 1200) equipped with a ZORBAX Eclipse plus C18 4.6 × 250 mm, 5 μm column was used for amino acid analysis. The mobile phase were A: 500 mL 20 mM sodium acetate + 2 mg EDTA + 0.018% triethylamine (v/v) adjusted to pH 7.2 with acetic acid + 0.3% tetrahydrofuran and B: 100 mL 20 mM sodium acetate adjusted to pH 7.2 with acetic acid + 200 mL methanol + 200 mL. The injection volume and flow rate were 0.45 mL/min and 1 μL. The separation gradient was: 0 min (100:0), 17 min (40:60), 18 min (0:100), 18.1 min (0:100), 23.9 min (0:100), 24 min (0:100), 25 min (100:0). The column was operated at 35°C and the wavelength was set at 254–340 nm. Amino acid standards was dissolved in methanol to give different concentration of 1, 5, 10, 15, 20 and 25 µg/mL. The linear regression equation were calculated with *Y* = *aX* ± *b*, where *X* was concentration of flavonoid and *Y* was the peak area of flavonoids obtained from UHPLC.

### 3.7. Antinutrient Analysis

About 1 g of freeze dried sample was homogenized with 0.1 M phosphoric acid (30 mL) and the solution was centrifuged at 8000 rpm for 20 min. Supernatant (3 mL) was transferred to a vial and 4 M sulfuric acid was added to each solution. All vials were heated to 100 °C for 50 min in order to hydrolyze them. After 50 min mixtures were cooled immediately in ice. The hydrolysis mixture was transferred to a Micro Dist tube containing 0.8 M MgCl_2_ (0.75 mL) and heated again for 45 min. After cooling to room temperature, cyanide and tannin were analyzed by ion chromatography (column: Guard: Ionpac AG7 40 × 250 mm; Fellow rate 1 mL/min; Injection volume 20 µL; 0.5 M sodium acetate/0.1 M sodium hydroxide/0.5% ethylenediamine. To prepare the standard solution standards were dissolved in HPLC grade methanol. The linear regression equation were calculated with *Y* = a*X* ± b, where *X* was concentration of compound and *Y* was the peak area of each compounds [49,50]. For phytic acid analysis spectrophotometric method was used. Stock solution of trichloroacetic acid (10 mL), hydrochloric acid (5 mL) and sulphuric acid (25 mL) was prepared. Plant samples (1 g) were extracted with 25 mL of stock solution at room temperature and constant shaking at medium speed in an orbital mixer in different length of extraction time. The mixture was centrifuged at 17,000 rpm for 30 min at 15 °C and the supernatants were collected and 0.5 mL of supernatant was mixed with 1 mL of ferric (III) chloride solution. The solution was heated for 30 min in a boiling water bath. Solutions were cooled at room temperature and centrifuged for 30 min at 4500 rpm. Then, 1 mL of the supernatant was transferred to another test tube and mixed with 2,2'-bipyridine. The absorbance of the reaction mixture was measured at 519 nm and distilled water was used as a blank. The method was calibrated with standard phytic acid solutions for each set of analysis [51].

### 3.8. Statistical Analysis

A split-split plot experiment based on randomized complete block design with three replications was employed. Factors were includes: CO_2_ concentrations (400 and 800 μmol/mol; main plot), varieties (H. Bara and H. Bentong; sub plot) and plant parts (leaf and rhizome; sub-sub plot). Data were subjected to an analysis of variance using Statistical Analysis System (SAS) version 9.0 (2002) and the means were compared using the Duncan’s multiple range test. Experimental results are present as means ± standard deviation of three replicates.

## 4. Conclusions

The results of the current study indicated that CO_2_ enrichment may be an effective method to increase ginger quality. Biochemical values except proteins were enhanced when young ginger was grown under double ambient CO_2_ concentration. The concentration of amino acid in the leaf of H. Bentong and H. Bara were changed significantly with CO_2_ treatment and differed depending on the plant part investigated. Initially, CO_2_ enrichment increased total soluble amino acids in H. Bentong and H. Bara leaves. Protein reduction was observed due to CO_2_ enrichment in both studied ginger varieties. Accordingly, physiological strategies and agronomic practices to mitigate changes in phytochemicals of crops and their quality should be a priority topic for further studies, which will be more and more related with food security. Generally, the CO_2_ enrichment technique is able to enhance young ginger quality based on amino acids and antinutrient content.

## Supplementary Materials

Supplementary materials can be accessed at: http://www.mdpi.com/1420-3049/19/10/16693/s1.

## Supplementary Files

Supplementary File 1
