# Supplementary material for: Changes in Nutritional Metabolites of Young Ginger (Zingiber officinale Roscoe) in Response to Elevated Carbon Dioxide
Source: Molecules. 2014 Oct 16;19(10):16693–706. doi: 10.3390/molecules191016693 (PMC6270952; doi:10.3390/molecules191016693)
Supplement: Supplementary File 1 [file molecules-19-16693-s001.pdf]

Supplementary Materials

ANOVA Tables of statistical analysis

Table S1. Total carbohydrate.

Source	DF	Anova SS	Mean Square	F Value	Pr > F
rep	2	1812.0475	906.02375	2.79	0.1207
CO_2	1	32841.20167	32841.20167	100.98	<0.0001
rep × CO_2	2	630.70583	315.35292	0.97	0.4197
var	1	1636.80167	1636.80167	5.03	0.0551
CO_2 × var	1	754.88167	754.88167	2.32	0.1661
rep × var (CO_2)	4	153.84667	38.46167	0.12	0.9722
part	1	15504.16667	15504.16667	47.67	0.0001
CO_2 × part	1	2448.24	2448.24	7.53	0.0253
Var × part	1	533.92667	533.92667	1.64	0.236
CO_2 × var × part	1	248.32667	248.32667	0.76	0.4077

Notes: R-Square:0.956026; Coeff Var:9.294641; Root MSE:18.03393; TSC Mean:194.0250.

Table S2. Protein.

Source	DF	Anova SS	Mean Square	F Value	Pr > F
rep	2	9.308575	4.6542875	10.93	0.0052
CO_2	1	190.4066667	190.4066667	446.99	<0.0001
rep × CO_2	2	2.0383583	1.0191792	2.39	0.1533
var	1	30.0608167	30.0608167	70.57	<0.0001
CO_2 × var	1	5.3016	5.3016	12.45	0.0078
rep × var (CO_2)	4	3.3958333	0.8489583	1.99	0.1886
part	1	284.41935	284.41935	667.7	<0.0001
CO_2 × part	1	8.0736	8.0736	18.95	0.0024
Var × part	1	16.9680167	16.9680167	39.83	0.0002
CO_2 × var × part	1	5.6842667	5.6842667	13.34	0.0065

Notes: R-Square:0.993905; Coeff Var:3.671295; Root MSE:0.652664; TSC Mean:17.77750.

Table S3. Sucrose.

Source	DF	Anova SS	Mean Square	F Value	Pr > F
rep	2	15.163333	7.581667	13.6	0.0027
CO_2	1	1152.320417	1152.320417	2066.94	<0.0001
rep × CO_2	2	0.563333	0.281667	0.51	0.6214
var	1	71.070417	71.070417	127.48	<0.0001
CO_2 × var	1	17.170417	17.170417	30.8	0.0005
rep × var (CO_2)	4	6.846667	1.711667	3.07	0.0829
part	1	255.45375	255.45375	458.21	<0.0001
CO_2 × part	1	8.760417	8.760417	15.71	0.0042
Var × part	1	5.320417	5.320417	9.54	0.0149
CO_2 × var × part	1	0.120417	0.120417	0.22	0.6545

Notes: R-Square:0.997099; Coeff Var:2.220273; Root MSE:0.746659; TSC Mean:33.62917.

Table S4. Glucose.

Source	DF	Anova SS	Mean Square	F Value	Pr > F
rep	2	11.954775	5.977387	5.92	0.0264
CO_2	1	1576.746704	1576.746704	1561.74	<0.0001
rep × CO_2	2	9.416608	4.708304	4.66	0.0454
var	1	4.208437	4.208437	4.17	0.0755
CO_2 × var	1	4.292604	4.292604	4.25	0.0731
rep × var (CO_2)	4	2.675483	0.668871	0.66	0.6353
part	1	504.625104	504.625104	499.82	<0.0001
CO_2 × part	1	0.362604	0.362604	0.36	0.5656
Var × part	1	80.923538	80.923538	80.15	<0.0001
CO_2 × var × part	1	6.273037	6.273037	6.21	0.0374

Notes: R-Square:0.996345; Coeff Var:4.714293; Root MSE:1.004793; TSC Mean:21.31375.

Table S5. Fructose.

Source	DF	Anova SS	Mean Square	F Value	Pr > F
rep	2	11.1610333	5.5805167	23.16	0.0005
CO_2	1	841.5872667	841.5872667	3493.03	<0.0001
rep × CO_2	2	5.2140333	2.6070167	10.82	0.0053
var	1	7.3926	7.3926	30.68	0.0005
CO_2 × var	1	11.0976	11.0976	46.06	0.0001
rep × var (CO_2)	4	1.0924	0.2731	1.13	0.4061
part	1	69.6322667	69.6322667	289.01	<0.0001
CO_2 × part	1	5.8806	5.8806	24.41	0.0011
Var × part	1	0.0112667	0.0112667	0.05	0.8342
CO_2 × var × part	1	9.5256	9.5256	39.54	0.0002

Notes: R-Square:0.998002; Coeff Var:3.557741; Root MSE:0.490850; TSC Mean:13.79667.

Table S6. Glutamine.

Source	DF	Anova SS	Mean Square	F Value	Pr > F
rep	2	9.6308333	4.8154167	3.34	0.0881
CO_2	1	581.1504167	581.1504167	403.23	<0.0001
rep × CO_2	2	2.6558333	1.3279167	0.92	0.4364
var	1	396.09375	396.09375	274.83	<0.0001
CO_2 × var	1	97.20375	97.20375	67.44	<0.0001
rep × var (CO_2)	4	2.77	0.6925	0.48	0.7501
part	1	324.8704167	324.8704167	225.41	<0.0001
CO_2 × part	1	298.9204167	298.9204167	207.4	<0.0001
Var × part	1	47.88375	47.88375	33.22	0.0004
CO_2 × var × part	1	95.6004167	95.6004167	66.33	<0.0001

Notes: R-Square:0.993829; Coeff Var:4.056384; Root MSE:1.200521; TSC Mean:29.59583.

Table S7. Histidine.

Source	DF	Anova SS	Mean Square	F Value	Pr > F
rep	2	5.905833	2.952917	2.56	0.1385
CO_2	1	322.666667	322.666667	279.37	<0.0001
rep × CO_2	2	0.515833	0.257917	0.22	0.8047
var	1	256.106667	256.106667	221.74	<0.0001
CO_2 × var	1	45.375	45.375	39.29	0.0002
rep × var (CO_2)	4	3.658333	0.914583	0.79	0.5622
part	1	1190.041667	1190.041667	1030.34	<0.0001
CO_2 × part	1	30.826667	30.826667	26.69	0.0009
Var × part	1	66.666667	66.666667	57.72	<0.0001
CO_2 × var × part	1	1.215	1.215	1.05	0.3351

Notes: R-Square:0.995218; Coeff Var:9.075659; Root MSE:1.074709; TSC Mean:11.84167.

Table S8. Glutamic acid.

Source	DF	Anova SS	Mean Square	F Value	Pr > F
rep	2	10.463333	5.231667	3.13	0.0989
CO_2	1	1612.120417	1612.120417	965.34	<0.0001
rep × CO_2	2	0.823333	0.411667	0.25	0.7872
var	1	519.870417	519.870417	311.3	<0.0001
CO_2 × var	1	0.570417	0.570417	0.34	0.575
rep × var (CO_2)	4	1.786667	0.446667	0.27	0.8909
part	1	571.350417	571.350417	342.13	<0.0001
CO_2 × part	1	245.120417	245.120417	146.78	<0.0001
Var × part	1	12.760417	12.760417	7.64	0.0245
CO_2 × var × part	1	9.75375	9.75375	5.84	0.0421

Notes: R-Square:0.995544; Coeff Var:4.015905; Root MSE:1.292285; TSC Mean:32.17917.

Table S9. Thrionine.

Source	DF	Anova SS	Mean Square	F Value	Pr > F
rep	2	19.623333	9.811667	14.89	0.002
CO_2	1	1356.006667	1356.006667	2058.45	<0.0001
rep × CO_2	2	0.143333	0.071667	0.11	0.8982
var	1	883.306667	883.306667	1340.88	<0.0001
CO_2 × var	1	79.206667	79.206667	120.24	<0.0001
rep × var (CO_2)	4	5.716667	1.429167	2.17	0.1631
part	1	2408.006667	2408.006667	3655.42	<0.0001
CO_2 × part	1	695.526667	695.526667	1055.83	<0.0001
Var × part	1	116.16	116.16	176.33	<0.0001
CO_2 × var × part	1	8.166667	8.166667	12.4	0.0078

Notes: R-Square:0.999055; Coeff Var:2.600003; Root MSE:0.811634; TSC Mean:31.21667.

Table S10. Lucine.

Source	DF	Anova SS	Mean Square	F Value	Pr > F
rep	2	11.9325	5.96625	15.45	0.0018
CO_2	1	1785.375	1785.375	4622.33	<0.0001
rep × CO_2	2	2.0575	1.02875	2.66	0.1299
var	1	3680.326667	3680.326667	9528.35	<0.0001
CO_2 × var	1	932.506667	932.506667	2414.26	<0.0001
rep × var (CO_2)	4	8.046667	2.011667	5.21	0.0231
part	1	337.5	337.5	873.79	<0.0001
CO_2 × part	1	1491.526667	1491.526667	3861.56	<0.0001
Var × part	1	487.801667	487.801667	1262.92	<0.0001
CO_2 × var × part	1	22.041667	22.041667	57.07	<0.0001

Notes: R-Square:0.999647; Coeff Var:1.392695; Root MSE:0.621490; TSC Mean:44.62500.

Table S11. Lysine.

Source	DF	Anova SS	Mean Square	F Value	Pr > F
rep	2	8.540833	4.270417	8.12	0.0119
CO_2	1	306.020417	306.020417	581.97	<0.0001
rep × CO_2	2	0.915833	0.457917	0.87	0.4548
var	1	1625.260417	1625.260417	3090.83	<0.0001
CO_2 × var	1	80.300417	80.300417	152.71	<0.0001
rep × var (CO_2)	4	5.476667	1.369167	2.6	0.1162
part	1	75.260417	75.260417	143.13	<0.0001
CO_2 × part	1	14.260417	14.260417	27.12	0.0008
Var × part	1	13.95375	13.95375	26.54	0.0009
CO_2 × var × part	1	32.43375	32.43375	61.68	<0.0001

Notes: R-Square:0.998058; Coeff Var:6.377225; Root MSE:0.725144; TSC Mean:11.37083.

Table S12. Valine.

Source	DF	Anova SS	Mean Square	F Value	Pr > F
rep	2	17.8425	8.92125	5.94	0.0262
CO_2	1	1703.535	1703.535	1134.74	<0.0001
rep × CO_2	2	10.0425	5.02125	3.34	0.088
var	1	250.906667	250.906667	167.13	<0.0001
CO_2 × var	1	85.881667	85.881667	57.21	<0.0001
rep × var (CO_2)	4	2.451667	0.612917	0.41	0.7982
part	1	750.401667	750.401667	499.85	<0.0001
CO_2 × part	1	12.906667	12.906667	8.6	0.0189
Var × part	1	1.041667	1.041667	0.69	0.429
CO_2 × var × part	1	0.06	0.06	0.04	0.8465

Notes: R-Square:0.995782; Coeff Var:3.701677; Root MSE:1.225255; TSC Mean:33.10000.

Table S13. Tyrosine.

Source	DF	Anova SS	Mean Square	F Value	Pr > F
rep	2	9.923333	4.961667	5.68	0.0292
CO_2	1	1199.920417	1199.920417	1372.64	<0.0001
rep × CO_2	2	2.063333	1.031667	1.18	0.3555
var	1	831.90375	831.90375	951.65	<0.0001
CO_2 × var	1	138.720417	138.720417	158.69	<0.0001
rep × var (CO_2)	4	6.113333	1.528333	1.75	0.2323
part	1	19.620417	19.620417	22.44	0.0015
CO_2 × part	1	51.920417	51.920417	59.39	<0.0001
Var × part	1	17.510417	17.510417	20.03	0.0021
CO_2 × var × part	1	2.220417	2.220417	2.54	0.1497

Notes: R-Square:0.996942; Coeff Var:4.268452; Root MSE:0.934969; TSC Mean:21.90417.

Table S14. Cyanide.

Source	DF	Anova SS	Mean Square	F Value	Pr > F
rep	2	324.72583	162.36292	7.22	0.0162
CO_2	1	35044.68375	35044.68375	1557.54	<0.0001
rep × CO_2	2	7.6225	3.81125	0.17	0.8471
var	1	17436.65042	17436.65042	774.96	<0.0001
CO_2 × var	1	1512.09375	1512.09375	67.2	<0.0001
rep × var (CO_2)	4	159.35833	39.83958	1.77	0.2278
part	1	77123.34375	77123.34375	3427.7	<0.0001
CO_2 × part	1	15145.35042	15145.35042	673.13	<0.0001
Var × part	1	8705.85042	8705.85042	386.93	<0.0001
CO_2 × var × part	1	161.72042	161.72042	7.19	0.0279

Notes: R-Square:0.998845; Coeff Var:2.971212; Root MSE:4.743416; TSC Mean:159.6458.

Table S15. Phytic acid.

Source	DF	Anova SS	Mean Square	F Value	Pr > F
rep	2	35.3725	17.68625	14.32	0.0023
CO_2	1	3.84	3.84	3.11	0.1159
rep × CO_2	2	2.0475	1.02375	0.83	0.4709
var	1	828.375	828.375	670.52	<0.0001
CO_2 × var	1	0.015	0.015	0.01	0.915
rep × var (CO_2)	4	1.67	0.4175	0.34	0.8452
part	1	3028.506667	3028.506667	2451.41	<0.0001
CO_2 × part	1	44.826667	44.826667	36.28	0.0003
Var × part	1	85.881667	85.881667	69.52	<0.0001
CO_2 × var × part	1	2.801667	2.801667	2.27	0.1705

Notes: R-Square:0.997556; Coeff Var:2.902070; Root MSE:1.111493; TSC Mean:38.30000.
